# Farmers’ and Dairymen’s Association

**Published:** 1882-04

**Authors:** 


### FARMERS' AND DAIRYMEN'S ASSOCIATION.

A meeting of the Farmers' and Dairymen's Association of Queens County was held in the hall of the Educational Association, in Westbury, Long Island, March 1st. The chief purpose of the meeting was to consider the subject of pleuro-pneumonia among cattle—a subject that has awakened a deep interest among the farmers of Long Island. A report was presented by a committee appointed at a previous meeting to investigate the measures that had been taken to prevent the spread of pleuro-pneumonia among cattle. The committee said that they had thoroughly examined into the subject of the inoculation of neat cattle, and were of the opinion that inoculation was the only prevention against the spread of contagious pleuro-pneumonia. A sub-committee of three had an interview with Governor Cornell, in which they urged the Governor to legalize inoculation. In relation to this, Governor Cornell said he was not thoroughly familiar with the subject, and, therefore, would have to take sufficient time to post himself before authorizing any action in the matter.

Mention was made by the chairman of the charges that diseased cattle had been sold to butchers.

A member of the association asked whether there was any penalty, under the State laws, where individual farmers inoculated their own cattle.

Mr. Williams answered that there was a law against the inoculation of cattle in this State, and if any farmer inoculated his cattle, the State Inspector was authorized to kill the animals.

President Cocks favored the drafting of a bill legalizing inoculation, which should be introduced in the Legislature at once. A resolution was adopted requesting the Queens County Agricultural Society to appoint a committee to unite with the legislative committee of the State Agricultural Association in securing legislation on the subject of inoculation for cattle. John D. Wing, Isaac H. Cocks, and General N. M. Curtis comprise a special committee, appointed by the State Agricultural Society, to attend to all legislation relating to the prevention of contagious diseases among cattle.

